# Mental Health, Resilience, and Physical Activity in Civilians Affected by Conflict-Related Trauma: A Cross-Sectional Study

**DOI:** 10.3390/healthcare13151781

**Published:** 2025-07-23

**Authors:** Gili Joseph

**Affiliations:** Science Faculty, Kibbutzim College of Education, 149 Derech Namir, Tel-Aviv 62507, Israel; gilijosephphd@gmail.com or gili.joseph@smkb.ac.il; Tel.: +972-544577095

**Keywords:** physical activity, resilience, well-being, anxiety, conflict-related trauma

## Abstract

**Background**: Mass casualty events in conflict-affected regions can lead to the displacement of civilians and are often accompanied by substantial psychological and emotional impact on those affected. While physical activity is known to support mental health, the ways in which it relates to anxiety, resilience, and well-being in conflict-affected populations are still being explored. Objective: This study examined the associations among physical activity, anxiety, resilience, and well-being in evacuees from a conflict-affected zone. We hypothesized that higher levels of intense physical activity would be associated with higher levels of resilience and well-being and lower levels of anxiety. **Methods**: In this cross-sectional study, 107 evacuees completed an online survey in December 2023. The questionnaire assessed the frequency and intensity of physical activity, generalized anxiety, resilience, and well-being. Participants were categorized by weekly total physical activity levels. Data was analyzed using ANOVA, Pearson correlations, and multiple linear regression. **Results**: Evacuees engaging in more than three hours of vigorous-intensity physical activity exhibited significantly higher resilience and better well-being compared to those with lower activity levels. Although not statistically significant, the data suggested a possible pattern of lower anxiety among evacuees engaging in higher levels of physical activity. Regression analysis identified higher resilience and lower anxiety as significant predictors of greater mental well-being. Additionally, residing in a community exposed to a higher number of traumatic events was associated with reduced well-being. The overall model explained a substantial portion of the variance in mental well-being. **Conclusions**: Physical activity, especially intense exercise, is associated with improved mental health and resilience among evacuees, supporting its inclusion in interventions for trauma-affected populations.

## 1. Introduction

Communities in a conflict-affected region of Israel have faced substantial disruptions to daily life, including large-scale displacement and relocation to temporary accommodations across the country. These circumstances have given rise to emotional distress, social dislocation, and psychological strain, highlighting the importance of examining the broader impacts of such disruptions on civilian well-being [1]. The absence of familiar frameworks and the challenges of adjusting to temporary living conditions led to heightened anxiety and sense of crisis [2,3]. These circumstances intensified the need for psychological and physical support systems.

Previous studies have indicated a strong connection between physical activity and improved mental health. Regular physical activity not only improves physical fitness but also serves as a means for emotional regulation and mood enhancement. This activity reduces cortisol levels and increases the secretion of endorphins, known as “happiness molecules”, due to their calming and uplifting effects [4]. Additionally, physical activity regulates serotonin and dopamine levels, two critical neurotransmitters responsible for feelings of relaxation, motivation, and satisfaction [5]. These effects are particularly significant for populations dealing with extreme stress or traumatic experiences. Individuals suffering from trauma tend to develop prolonged physiological responses to stress, such as heightened sympathetic nervous system activation [6]. Physical activity provides natural means of regulating these systems, reducing anxiety levels, and improving sleep quality, which is often impaired following exposure to traumatic events. Furthermore, engaging in physical activity helps to alleviate feelings of social isolation and depression. Whether through independent activities (e.g., running alone) or group activities (e.g., yoga or team sports), physical interaction with the environment fosters a sense of belonging and social resilience, both of which are essential for mental health [7].

Mental resilience is defined as an individual’s ability to cope with stress, adapt to it, and grow from it [8]. In this context, physical activity plays a key role in strengthening personal resilience. First, it provides a sense of control, which is critical in traumatic life situations where individuals experience a loss of control over their circumstances. Regaining a sense of control through purposeful and planned activities, such as strength training or aerobic exercise, helps trauma survivors restore confidence in their ability to handle challenges [9]. Second, physical activity contributes to the improvement of coping abilities. Studies suggest that individuals who engage in regular physical activity develop higher mental resilience because they practice dealing with physical challenges, which translates into better psychological coping skills [10,11].

One of the most significant aspects of physical activity is its contribution to reducing anxiety. Studies show that aerobic exercise, such as running, brisk walking, cycling, and swimming, significantly reduce symptoms of anxiety [12,13]. This effect stems from several factors: first, the activity itself distracts from negative thoughts and provides external focus. Second, it generates a sense of achievement and self-efficacy, which are crucial components of anxiety reduction and self-confidence enhancement. Moreover, physical activity helps regulate the sympathetic nervous system, responsible for the body’s response to stress. Physical activity helps “train” the body to respond more balanced to stress, thereby reducing the negative physiological effects of anxiety [14]. Particularly, populations that have experienced trauma tend to develop symptoms of post-traumatic stress disorder (PTSD), which includes hyperarousal, sleep disturbances, and intrusive memories. Previous research has indicated that physical activity helps to alleviate these symptoms through neurobiological mechanisms of emotional regulation and enhancing mental resilience [15]. Furthermore, participating in group activities can provide emotional support and strengthen the sense of belonging, which is crucial in coping with traumatic experiences [16].

Previous studies have shown that intervention programs that incorporate physical activity have been effective in reducing anxiety, improving mood, and enhancing mental resilience [17,18,19,20]. Structured programs combining strength training, yoga, group running, or walks can provide effective tools for emotional regulation and increased personal control. Participants who engage in physical activity experience diverse positive effects that go beyond symptom reduction; they also improve crucial areas such as family and social relationships, daily functioning, self-confidence, and personal empowerment. Participants view physical activity as treatment that improves mental and physical distress, including symptoms related to PTSD and high levels of general stress (such as anxiety, mood changes, fatigue, pain, headaches, and sleep problems). The sense of well-being during physical activity is considered a significant health outcome, contributing to life quality [18,19,20]. Additionally, the positive perception of physical activity can enhance essential self-regulation processes necessary for perseverance in treatment and overall motivation [21].

The present study aims to examine the associations between physical activity, anxiety, resilience, and well-being among evacuees from a conflict-affected zone during their stay in temporary evacuation settings. We hypothesized that higher levels of physical activity would be associated with higher levels of resilience and well-being and lower levels of anxiety.

## 2. Materials and Methods

### 2.1. Study Population and Design

This study included individuals aged 25 years and older who were residents of rural communities in a conflict-affected region of southern Israel. The mean age of participants was 46.2 years (SD = 12.9). Of the 107 participants, 77 (72.0%) were female and 30 (28.0%) were male. In terms of community origin, 43 participants (40.2%) were evacuated from “Community A”, which was directly exposed to severe trauma and violence during the conflict. An additional 19 participants (17.8%) were from “Community B,” a nearby locality within the same region that was not directly impacted by the traumatic events. The remaining 45 participants (42.1%) were evacuees from nine other rural communities within the broader conflict zone. Marital status was reported as follows: 85 participants (79.4%) were married or in a committed relationship, 8 (7.5%) were single, 9 (8.4%) were divorced, 4 (3.7%) were widowed, and 1 (0.9%) reported another status. Regarding education level, 22 participants (20.6%) had completed high school, 55 (51.3%) held a bachelor’s degree, 29 (27.1%) had a master’s degree or higher, and 1 participant (0.9%) reported no formal education. A detailed breakdown of demographic characteristics is presented in Table 1.

Inclusion criteria required participants to be at least 25 years old, to have been displaced from their homes due to the conflict, and to provide informed consent. Individuals younger than 25 or those not evacuated from their homes were excluded.

A cross-sectional correlational design was employed. Data collection occurred approximately two months after the onset of the conflict (December 2023), while evacuees were living in temporary accommodations such as hotels, kibbutzim, and guesthouses across the country. Members of the research team approached evacuees in person at these sites, invited them to participate, and provided a link to a digital questionnaire (via Google Forms). Participants completed the questionnaire in the presence of the researchers. No online recruitment or snowball sampling methods were used; all participants were recruited directly and individually on site.

### 2.2. Questionnaire and Variables

The questionnaire comprised several sections.

The demographic section collected data on age, gender, marital status, education level, place of residence during the conflict, current residence, employment status prior to the conflict, whether family members had been drafted or affected by the conflict, and whether the current residence included a protected shelter.

Anxiety symptoms were measured using the Generalized Anxiety Disorder Screener (GAD-7) [22], a validated 7-item instrument assessing symptom severity over the past two weeks. Items were rated on a 4-point Likert scale (1 = never to 5 = nearly every day). The GAD-7 is widely used in clinical and research settings and has been validated in Hebrew (Cronbach α = 0.931); it is also recommended by the Israeli Ministry of Health’s Professional Committee for Clinical Psychology.

Resilience was assessed with the Connor-Davidson Resilience Scale (CD-RISC) [23], which evaluates an individual’s ability to adapt to adversity. Responses were rated on a 6-point Likert scale (1 = never to 6 = nearly all the time). The Hebrew version of the CD-RISC was translated and validated by the original developers (Cronbach α = 0.923) and has been widely used in Israeli research, demonstrating strong psychometric properties.

Well-being was measured using a 10-item well-being questionnaire [24] assessing emotional, psychological, and social aspects of well-being. Participants responded on a 5-point Likert scale (1 = agree to 5 = agree very much). The Hebrew version, adapted and validated by Florian and Drori (Cronbach α = 0.96) [25], has been shown to have good reliability and validity in the Israeli population.

Physical activity was assessed using the International Physical Activity Questionnaire—Short Form (IPAQ-SF), a widely used and validated self-report instrument developed for international comparison of physical activity levels [26]. The questionnaire was administered in its Hebrew-translated version, which has previously demonstrated acceptable validity and reliability in a study among Israeli undergraduate students [27].

Participants reported their average weekly duration of physical activity (in minutes) and the amount of time spent in vigorous-intensity exercise, defined as activities inducing heavy breathing and a noticeably elevated heart rate, as recommended by the World Health Organization (WHO) [28].

For analysis, physical activity was categorized in two ways:

Total weekly exercise duration: Non-exercisers: 0–30 min/week, Moderate exercisers: 31–180 min/week, High exercisers: >180 min/week.

Vigorous-intensity activity duration: Non-exercisers: 0–30 min/week, Moderate exercisers: 31–180 min/week, High exercisers: >180 min/week.

### 2.3. Statistical Analysis

Descriptive statistics (means, standard deviations, and frequencies) were calculated to summarize demographic characteristics and key study variables. Only fully completed questionnaires were included in the analysis, therefore, there were no missing data to address.

Normality of the continuous variables (resilience, anxiety, and well-being) was assessed using skewness and kurtosis values. All values fell within acceptable ranges (|skewness| < 1, |kurtosis| < 3), indicating that the assumption of approximate normality was met across the three physical activity groups (non-exercisers, moderate exercisers, and high exercisers). Homogeneity of variances was tested using Levene’s test, which indicated that the assumption was met for resilience (*p* = 0.787) and well-being (*p* = 0.526), but not for anxiety (*p* = 0.002). Therefore, in addition to the one-way ANOVA, a non-parametric Kruskal–Wallis test was conducted for anxiety.

To examine group differences in psychological variables across levels of physical activity (in terms of total duration and intensity), one-way analyses of variance (ANOVA) were conducted for resilience and well-being, as the assumptions of normality and homogeneity of variances were met. For anxiety, the Kruskal–Wallis test was used due to violation of the homogeneity of variances assumption. Pearson correlation coefficients were computed to assess associations among anxiety, resilience, well-being, and physical activity variables. To evaluate the predictive value of physical activity and psychosocial factors on mental well-being, a multiple linear regression analysis was performed. Predictor variables included weekly physical activity duration, frequency of physical activity, duration of vigorous-intensity activity, anxiety levels, resilience levels, perceived level of security in the current residence, and place of residence. Place of residence was categorized as “Community A” (high exposure to trauma and violence) and “Community B” (a nearby area not directly impacted by the conflict).

A post hoc power analysis was conducted to evaluate whether the sample size (N = 107; group sizes *n* = 22, 45, and 40) provided sufficient statistical power for the primary analyses. Based on the observed group means and standard deviations, effect sizes were estimated as follows: anxiety (Cohen’s d = 0.32, small to medium), resilience (d = 0.71, medium to large), and well-being (d = 0.92, large). These values indicate that the study had adequate statistical power (≥0.80) to detect medium to large effects using both ANOVA and regression models. All statistical tests were two-tailed, and statistical significance was set at *p* < 0.05.

All statistical analyses were conducted using IBM SPSS Statistics, version 29.0.1.0.

Ethical approval for this study was granted by the institutional ethics committee (approval number: ETD1520252).

## 3. Results

The study sample consisted of 107 participants, predominantly women, with an average age of 46.2 years. Most participants were married or in long-term relationships, and more than three-quarters had post-secondary education. The majority were employed prior to the conflict. Approximately 40% had resided in “Community A,” which was heavily affected by the events of 7 October, while others were from “Community B” or other nearby communities within the conflict zone. Following evacuation, participants were housed in various temporary settings, including hotels, kibbutzim, and private residences. A considerable portion of respondents reported having close family members serving in the military reserves, and more than half had someone from their immediate social circle harmed during the initial outbreak of violence. Access to protected shelters in current accommodations varied, with around 60% indicating they had such protection. Full demographic details are presented in Table 1.

A series of statistical tests were conducted to examine whether differences existed in resilience, anxiety, and well-being among participants grouped by total weekly physical activity duration: non-exercisers (0–30 min/week, *n* = 22), moderate exercisers (31–180 min/week, *n* = 45), and high exercisers (>180 min/week, *n* = 40).

**Figure 1:**
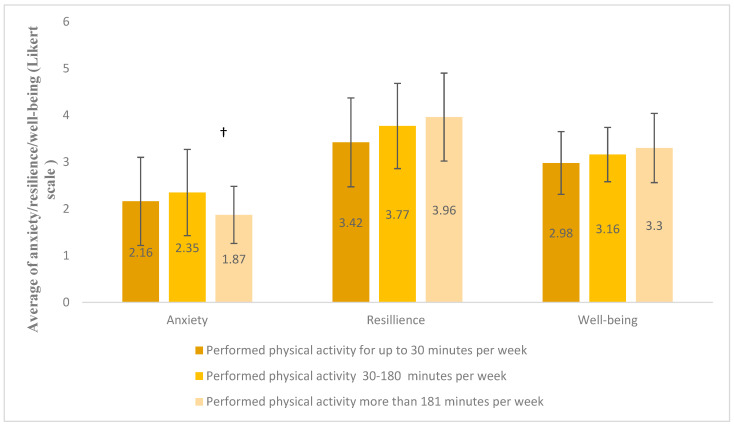
The differences in the levels of anxiety, resilience, and well-being between the groups according to the weekly time of physical activity (minutes). A Kruskal–Wallis test was conducted for anxiety due to unequal variances across groups, while one-way ANOVA tests were used for resilience and well-being. († *p* = 0.075).

One-way ANOVA tests revealed no significant differences between the groups in resilience or well-being. However, for anxiety, Levene’s test indicated a violation of the homogeneity of variances assumption (*p* = 0.002). Therefore, a Kruskal–Wallis test was used instead, yielding a marginally significant result (H = 5.026, *p* = 0.081). Pairwise comparisons showed that participants in the high activity group reported lower anxiety levels compared to the moderate group (1.95 ± 0.70 vs. 2.21 ± 0.87), with an unadjusted significance level of *p* = 0.025, though this did not remain significant after Bonferroni correction (*p* = 0.075). No significant differences were found when comparing the “non-exercisers” group to others (Figure 1).

Differences in anxiety, resilience, and well-being were examined based on participants’ weekly duration of vigorous physical activity, defined as activity that caused heavy breathing. Participants were grouped into three categories: non-exercisers (0–30 min per week, *n* = 24), moderate exercisers (31–180 min per week, *n* = 60), and high exercisers (more than 180 min per week, *n* = 23).

**Figure 2:**
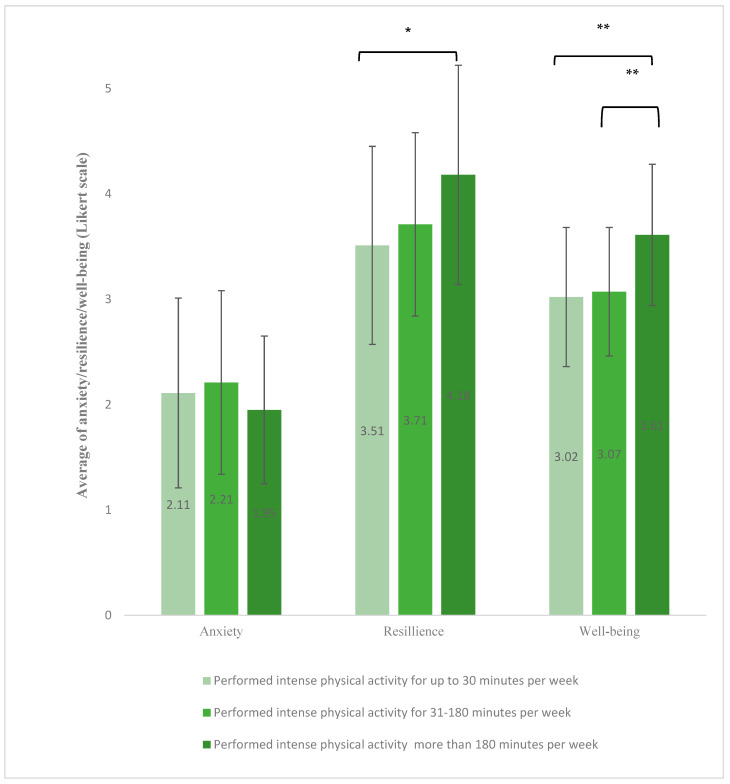
The differences in the levels of anxiety, resilience, and well-being between the three groups, according to the weekly time of intense physical activity (in minutes), which caused participants to breathe heavily. An ANOVA test was performed to find the differences between the groups (* = *p* < 0.05, ** = *p* < 0.005). Kruskal–Wallis test was used for anxiety.

Analysis of variance (ANOVA) indicated significant group differences in both resilience and well-being. Participants in the high-intensity group reported significantly higher resilience scores than non-exercisers (mean = 4.18 ± 1.04 vs. 3.51 ± 0.94, *p* < 0.05). Well-being scores were also significantly higher in the high exercise group compared to both moderate and non-exercisers (mean = 3.61 ± 0.67 vs. 3.07 ± 0.61 and 3.02 ± 0.66, respectively; *p* < 0.005). A Kruskal–Wallis test was used for anxiety, no significant differences were observed between the groups in anxiety levels based on the duration of intense physical activity (Figure 2).

The Pearson’s correlation coefficient measurement indicated a small to moderate but statistically significant positive association between resilience levels and the total weekly physical activity level (r = 0.300, *p* = 0.002), as well as between resilience levels and weekly exercise duration involving vigorous-intense physical activity causing heavy breathing (r = 0.296, *p* = 0.002), suggesting that as the total exercise time and time spent on vigorous-intense physical activities causing heavy breathing increased, resilience levels also rose (Table 2).

Similarly, a small to moderate but statistically significant positive association was found between well-being levels and weekly exercise duration (r = 0.264, *p* = 0.006), as well as between well-being and time spent on vigorous-intense physical activity (r = 0.312, *p* = 0.001), indicating that longer exercise durations and higher-intensity exercise were associated with higher levels of well-being and resilience.

Regarding anxiety, a small but statistically significant negative correlation was observed between the time spent on physical activity per week and anxiety levels (r = −0.199, *p* = 0.04), indicating that, as the duration of physical activity increased, anxiety levels decreased. We also found a small to moderate negative correlation between anxiety levels and resilience (r = −0.33, *p* < 0.001), as well as between anxiety levels and well-being (r = −0.473, *p* < 0.001). In other words, as resilience and well-being increased, anxiety levels decreased. Finally, a moderate positive association was found between resilience and well-being (r = 0.553, *p* < 0.001), indicating that as resilience levels increased, so did well-being levels.

Table 3 presents the results of a multiple regression analysis conducted to predict the well-being of the population studied. The constant coefficient was 2.218 (*p* < 0.001), indicating a significant baseline value for well-being when all predictors were held constant. The regression analysis indicates that 44% of the variance in participants’ well-being levels were explained by resilience, anxiety, intense physical activity duration, and residence in “Community A” (R^2^= 0.482, R = 0.695). Anxiety levels (B = −0.217, *p* = 0.002) and resilience levels (B = 0.298, *p* < 0.001) were both significantly associated with mental well-being, higher resilience, and lower anxiety levels.

Residence in “Community A”, a location that experienced particularly high levels of trauma and direct exposure to violence (B = −0.289, *p* = 0.012) was found to have a significant negative association with mental well-being, whereas residence in “Community B”, an area within the region that was not directly impacted by the traumatic events (B = 0.060, *p* = 0.672), was not a significant predictor. Weekly duration of intense physical activity (B = 0.066, *p* = 0.058) exhibited a positive trend, suggesting a possible positive impact on mental well-being, though the result was non-significant.

## 4. Discussion

This study examined the relationship between physical activity, anxiety, resilience, and well-being among individuals affected by a large-scale traumatic event in a conflict-affected area. The findings suggest that physical activity may support psychological health during times of crisis, particularly through its association with enhanced resilience and well-being. While anxiety levels were lower among participants who reported higher levels of physical activity, this association did not reach statistical significance and should be interpreted as a potential trend rather than a confirmed effect.

Importantly, the analyses revealed that the intensity of physical activity—specifically, the time spent engaging in vigorous-intensity activity that induces heavy breathing—was more consistently associated with psychological benefits than total weekly activity duration. Significant positive associations were observed between vigorous activity and both resilience and well-being, while the relationship with anxiety remained non-significant. These findings indicate that not all forms of physical activity may contribute equally to mental health outcomes, and that higher-intensity exercise might offer more pronounced psychological advantages. This pattern aligns with previous studies suggesting that the physiological demands of vigorous activity may more strongly stimulate endorphin release, modulate autonomic responses, and enhance mood-regulating processes [14,29,30]. Psychosocial mechanisms, including improved self-efficacy, social interaction, and mastery, also offer relevant explanatory frameworks [31,32,33].

While most studies support these benefits, some research has found more nuanced patterns. For example, Eime et al. [34] reported that women who participated in club sports showed better mental health and life satisfaction compared to those who engaged in gym-based activities or walking. This suggests that the social context in which physical activity occurs may play a pivotal role in enhancing mental well-being. Similarly, Cekin [35] found that regular physical activity was associated with higher levels of self-esteem, optimism, and happiness in emerging adults, although the magnitude of these effects varied depending on individual factors and consistency of engagement.

The absence of a significant association between vigorous activity and anxiety in our study, despite the observable trend, contrasts with some literature emphasizing the anxiolytic effects of intense exercise [32]. This discrepancy may reflect individual differences in how people experience and tolerate intense activity, or it may point to a nonlinear relationship between physical exertion and anxiety modulation. It is also possible that other unmeasured factors, such as perceived control, fitness level, or baseline psychological state, moderated this association.

Regarding resilience, participants who engaged in higher levels of vigorous physical activity demonstrated significantly greater resilience, supporting earlier findings linking exercise to psychological adaptability and emotion regulation [4,5,9,10,15,33,36]. The moderate positive correlation between resilience and well-being is consistent with conceptual frameworks that view resilience as a buffer against stress, enabling emotional stability in the face of adversity [8,32,37]. Moreover, the observed negative associations between anxiety and both resilience and well-being reaffirm existing evidence regarding resilience’s role in protecting mental health [4,5,8,32,38].

Nevertheless, it is important to acknowledge that the literature on sport-based interventions presents a mixed picture. Janković et al. [39], for example, highlight the variability in psychological outcomes related to sport participation and suggest that the effectiveness of such interventions may depend on contextual and individual factors. These findings emphasize the complexity of designing physical activity-based strategies for trauma-affected populations.

The results point to both physiological and psychosocial pathways through which physical activity may influence psychological outcomes. Endorphin and dopamine release during exercise are known to elevate mood and reduce symptoms of anxiety and depression [33]. Simultaneously, physical activity may foster resilience by regulating physiological stress responses and supporting endocrine balance [4,5,15]. Additionally, structured activity routines promote discipline, goal-setting, and perseverance, all of which contribute to psychological growth following trauma [4,5,9,15,33,36,37].

The differences observed in mental well-being between communities provide further insight into how environmental context and trauma exposure shape psychological outcomes. Participants from Community A—who experienced direct exposure to violence—reported significantly lower well-being levels than those from Community B, who were also evacuated but not exposed to the same level of threat. This highlights the importance of both subjective and objective exposure to trauma as determinants of mental health. Moreover, access to protected shelters appeared to play a role: approximately 40% of participants lacked such access, potentially increasing their vulnerability and psychological distress. These findings underscore the complex interplay between internal coping resources and external conditions such as safety, support, and the degree of exposure to traumatic events.

While these findings offer valuable insights, they must be interpreted considering several methodological limitations. First, the cross-sectional design precludes causal inference and captures psychological states at a single point—approximately two months post-event—while mental health trajectories may evolve over time. Second, convenience sampling—necessitated by the dispersed nature of evacuees and the urgent post-crisis context—may introduce selection bias and limit generalizability. Recruitment occurred in temporary evacuee hotels under emotionally charged and logistically constrained conditions, which influenced participation willingness and contributed to a relatively small overall sample size.

In addition, uneven distribution across key subgroups should be acknowledged. Specifically, participant representation from different communities was imbalanced, with a higher proportion from Community A (directly affected) and fewer from Community B and other rural areas. Likewise, the distribution across physical activity levels was unequal, with notably fewer participants in the “no physical activity” group. These disparities may reduce statistical power and affect the generalizability of subgroup comparisons.

Furthermore, unmeasured confounding variables, such as individual coping strategies, social support, and pre-existing mental health conditions, could influence the associations observed. Ethical and emotional considerations prevented the collection of detailed psychological history, as participant well-being and minimizing retraumatization were prioritized. As a result, potential confounders could not be controlled for, which may have affected internal validity.

Despite these limitations, several strengths enhance the value of this study. The sample included a relatively diverse demographic profile in terms of gender, marital status, education, and occupational backgrounds, allowing for a broader understanding of physical activity’s role across different population segments. Additionally, this is one of the first studies to explore the interplay of physical activity, resilience, and mental health specifically among civilians displaced by conflict in this region, providing novel insights with potential international relevance.

Future research should employ longitudinal designs to monitor changes in physical activity, resilience, and psychological health over time, helping to establish causal pathways. Additionally, examining potential mediators such as social support, coping mechanisms, and personality traits could clarify how physical activity exerts its effects. Intervention studies integrating physical activity with psychosocial therapies are warranted to evaluate efficacy in improving mental health outcomes in trauma-affected populations. Confirmatory studies should also explore optimal types, durations, and intensities of physical activity to maximize resilience and well-being benefits. These efforts will help inform tailored intervention strategies and public health policies in conflict and disaster settings.

## 5. Conclusions

This study highlights the potential psychological benefits of physical activity among civilians affected by traumatic events. A high volume of vigorous-intensity physical activity was associated with enhanced resilience (*p* = 0.035, d = −0.68) and well-being (*p* = 0.007, d = −0.89), with individuals engaging in higher levels of activity reporting better psychological outcomes. Although the association between physical activity and anxiety did not reach conventional statistical significance (*p* = 0.075), a trend toward lower anxiety was observed among the most active participants (d = 0.31). These findings suggest that promoting physical activity may contribute meaningfully to psychological recovery following trauma. Integrating physical activity into post-trauma intervention programs, alongside psychosocial support, could help address the complex needs of trauma-exposed communities and support improved long-term mental health outcomes.

## Figures and Tables

**Table 1:** Demographic data of the study population.

Characteristic	Categories	Frequency (n)	Percentage (%)
Age (years)	Mean ± SD	46.2 ± 12.9	–
Gender	Female	77	72.0%
	Male	30	28.0%
Marital status	Married/Committed Relationship	85	79.4%
	Single	8	7.5%
	Divorced	9	8.4%
	Widowed	4	3.7%
	Other	1	0.9%
Education level	High School	22	20.6%
	Bachelor’s Degree	55	51.3%
	Master’s Degree or Higher	29	27.1%
	None	1	0.9%
Place of residence at time of conflict	“Community A”	43	40.2%
	“Community B”	19	17.8%
	Other communities	45	42.1%
Post-evacuation residence	Evacuation Hotel (Eilat/Dead Sea/Tel Aviv)	49	46%
	Hosting Kibbutz	17	16.0%
	Temporary Rental	8	8.0%
	With Family	9	8.0%
	Other	34	22.0%
Employment before conflict	Employed	77	72.0%
	Self-Employed	15	14.0%
	Unemployed	3	2.8%
	Other	12	11.2%
Family members drafted	Combat Role	30	28.0%
	Support Role	15	14.0%
	Not Drafted	62	58.0%
Family members affected from the conflict	Yes	62	58.0%
	No	45	42.0%
Protected shelter in current residence	Yes	66	61.7%
	No	41	38.3%

Abbreviation: SD, standard deviation.

**Table 2:** Pearson correlation coefficients for the relationships between weekly physical activity duration, weekly intense physical activity duration (causing heavy breathing), and levels of resilience, well-being, and anxiety.

Variable	Weekly Intense Physical Activity Duration (Causing Heavy Breathing)	Weekly Physical Activity Duration	Well-Being	Anxiety	Resilience
Weekly intense physical activity duration (causing heavy breathing)	1				
Weekly physical activity duration	0.840 **	1			
Well-being	0.312 **	0.264 **	1		
Anxiety	−0.128	−0.199 *	−0.473 **	1	
Resilience	0.296 **	0.300 **	0.553 **	−0.33 **	1

* *p* < 0.05, ** *p* < 0.01. Bold numbers emphasize significance.

**Table 3:** Regression analysis for predicting mental well-being.

Predictor Variable	Unstandardized Coefficients (B)	Standard Error	Standardized Coefficients (Beta)	t	Sig.
(Constant)	2.218	0.466	-	4.760	<0.001
Current security levels	0.031	0.045	0.057	0.702	0.484
Weekly physical activity duration	−0.046	0.027	−0.251	−1.718	0.089
Weekly frequency of physical activity	0.238	0.300	0.104	0.792	0.430
Weekly duration of intense physical activity (causing heavy breathing)	0.066	0.034	0.291	1.918	0.058
Anxiety levels	−0.217	0.066	−0.273	−3.266	0.002
Resilience levels	0.298	0.058	0.421	5.166	<0.001
Residence in “Community A”	−0.289	0.113	−0.214	−2.569	0.012
Residence in “Community B”	0.060	0.141	0.035	0.425	0.672
Model quality indicators	Adjusted R square	R Square	R		
Value	0.440	0.482	0.695		

## Data Availability

Due to privacy or ethical restrictions, data will be available upon request from the author.
